# Predicting the Emergence of Major Neurocognitive Disorder Within Three Months After a Stroke

**DOI:** 10.3389/fnagi.2021.705889

**Published:** 2021-08-16

**Authors:** Eva Birgitte Aamodt, Till Schellhorn, Edwin Stage, Apoorva Bharthur Sanjay, Paige E. Logan, Diana Otero Svaldi, Liana G. Apostolova, Ingvild Saltvedt, Mona Kristiansen Beyer

**Affiliations:** ^1^Institute of Clinical Medicine, University of Oslo, Oslo, Norway; ^2^Division of Radiology and Nuclear Medicine, Oslo University Hospital, Oslo, Norway; ^3^Department of Neurology, School of Medicine, Indiana University, Indianapolis, IN, United States; ^4^Department of Neuromedicine and Movement Science, Faculty of Medicine and Health Science, NTNU – Norwegian University of Science and Technology, Trondheim, Norway; ^5^Department of Geriatrics, Clinic of Medicine, St. Olavs Hospital, Trondheim University Hospital, Trondheim, Norway

**Keywords:** stroke, neurocognitive disorders, dementia, rapid onset, prediction, MRI

## Abstract

**Background:** Neurocognitive disorder (NCD) is common after stroke, with major NCD appearing in about 10% of survivors of a first-ever stroke. We aimed to classify clinical- and imaging factors related to rapid development of major NCD 3 months after a stroke, so as to examine the optimal composition of factors for predicting rapid development of the disorder. We hypothesized that the prediction would mainly be driven by neurodegenerative as opposed to vascular brain changes.

**Methods:** Stroke survivors from five Norwegian hospitals were included from the “Norwegian COgnitive Impairment After STroke” (Nor-COAST) study. A support vector machine (SVM) classifier was trained to distinguish between patients who developed major NCD 3 months after the stroke and those who did not. Potential predictor factors were based on previous literature and included both vascular and neurodegenerative factors from clinical and structural magnetic resonance imaging findings. Cortical thickness was obtained via FreeSurfer segmentations, and volumes of white matter hyperintensities (WMH) and stroke lesions were semi-automatically gathered using FSL BIANCA and ITK-SNAP, respectively. The predictive value of the classifier was measured, compared between classifier models and cross-validated.

**Results:** Findings from 227 stroke survivors [age = 71.7 (11.3), males = (56.4%), stroke severity NIHSS = 3.8 (4.8)] were included. The best predictive accuracy (AUC = 0.876) was achieved by an SVM classifier with 19 features. The model with the fewest number of features that achieved statistically comparable accuracy (AUC = 0.850) was the 8-feature model. These features ranked by their weighting were; stroke lesion volume, WMH volume, left occipital and temporal cortical thickness, right cingulate cortical thickness, stroke severity (NIHSS), antiplatelet medication intake, and education.

**Conclusion:** The rapid (<3 months) development of major NCD after stroke is possible to predict with an 87.6% accuracy and seems dependent on both neurodegenerative and vascular factors, as well as aspects of the stroke itself. In contrast to previous literature, we also found that vascular changes are more important than neurodegenerative ones. Although possible to predict with relatively high accuracy, our findings indicate that the development of rapid onset post-stroke NCD may be more complex than earlier suggested.

## Introduction

Stroke is the second most frequent cause of death worldwide and a major cause of disability related to motor, cognitive, and behavioral impairments (Pendlebury, 2012). The last decade has seen major improvements in the treatment of stroke and survival rates are on the rise. With increased longevity and an aging population, stroke and dementia will consequently constitute a substantial part of the societal health burden in the years to come (Murray et al., 2012). Mild or major neurocognitive disorder (NCD) (previously “mild cognitive impairment” and “dementia,” respectively) is common after a stroke (American Psychiatric Association, 2013; Sachdev et al., 2014), with major NCD appearing in about 10% of survivors of a first-ever stroke (Pendlebury and Rothwell, 2019) and in about 30% of recurrent strokes (Mellon et al., 2015). The risk of developing major NCD is highest shortly after a stroke (Mok et al., 2017), with 10–20% of patients being diagnosed within the first year (Ihle-Hansen et al., 2011; Pendlebury, 2012). However, the risk remains elevated for months and years after the stroke (Mok et al., 2017), with the cumulative incidence increasing at a rate of 3% per year, starting after the immediate post-stroke period (Pendlebury, 2012).

Multiple risk factors for post-stroke NCD have been identified, including older age, lower education, pre-stroke disability, and pre-stroke major NCD. Factors related to the stroke lesion itself, such as the location (left hemisphere), stroke lesion volume, clinical stroke severity, and presence of early post-stroke complications such as seizures and delirium, have also been found to be important predictors (Pendlebury, 2012). Risk factors also include factors that are associated with vascular disease, such as diabetes, atrial fibrillation (AF), and white matter hyperintensities (WMHs). Neurodegenerative factors, such as global cortical atrophy and medial temporal lobe atrophy (MTA), are also associated with an increased risk (Pendlebury, 2009; Casolla et al., 2019). These vascular and neurodegenerative changes seem to interact, resulting in cumulative brain damage and cognitive decline (Schneider et al., 2009).

Post-stroke NCD is complex and the underlying mechanisms remain unclear beyond the fact that both neurodegenerative and vascular mechanisms seem to contribute to the cognitive decline (Thiel et al., 2014). In a review, Mok et al. (2017) hypothesized that post-stroke NCD patients may differ in disease etiology, as neurodegeneration-driven major NCD, caused for instance by Alzheimer’s Disease (AD), typically occurs relatively soon after a stroke (<6 months), whereas vascular-driven major NCD often develops later.

In the current study we aimed to investigate the ability of clinical- and imaging features to predict rapid development (<3 months) of major NCD after a stroke. Based on previous literature, we hypothesized that rapid onset post-stroke major NCD would be more strongly associated with neurodegenerative rather than vascular brain changes.

## Materials and Methods

### Nor-COAST

The current study is based on data from the Norwegian Cognitive Impairment After Stroke study (Nor-COAST) – a prospective longitudinal multicenter cohort study recruiting patients hospitalized with acute stroke at five Norwegian stroke units (Thingstad et al., 2018). Patient recruitment started in May of 2015 and was completed in March of 2017. Details of the Nor-COAST study are described elsewhere (Thingstad et al., 2018). The study was approved by the regional committee for medical and health research, REK Nord (REK number: 2015/171), and registered on clinicaltrials.gov (NCT02650531). REK Nord has also approved this current sub study (REK number: 2019/397). All participants provided written informed consent in accordance with the Declaration of Helsinki. If a potential participant was unable to give consent, written informed consent for participation was given by a family proxy. Participants signed a separate informed consent to partake in the MRI sub study.

### Subjects

Inclusion criteria for Nor-COAST: (a) patients admitted with acute ischemic or hemorrhagic stroke hospitalized within 1 week after onset of symptoms, diagnosed according to the World Health Organization (WHO) criteria; (b) age over 18 years; and (c) fluent in a Scandinavian language.

Exclusion criteria for Nor-COAST: (a) not treated in the participating stroke units; (b) symptoms explained by other disorders than ischemic brain infarcts or intracerebral hemorrhages; and (c) expected survival less than 3 months after stroke.

Inclusion criteria for MRI sub study: (a) patient included in Nor-COAST; (b) modified Rankin scale < 5 before the stroke; and (c) able to cooperate during MRI.

Exclusion criteria for MRI sub study: (a) severe functional impairment making MRI impossible to perform; (b) medical contraindications for MRI like claustrophobia or pacemaker; and (c) patient declining participation in MRI.

Further, some patients were excluded from the current sub-study due to missing a positive DWI of an acute stroke, and/or cognitive testing at 3 months. Missing a positive DWI was due to late execution of the study-specific MRI, typically >7 days after the acute stroke, making the acute stroke no longer visible on the DWI series.

### MRI Acquisition

A study-specific brain MRI was performed as soon as an MRI-machine was available during the acute/subacute phase of the stroke. Brain scans were acquired at five different hospitals, using a single MRI-scanner at each site (GE Discovery MR750, 3T; Siemens Biograph_mMR, 3T; Philips Achieva dStream, 1.5T; Philips Achieva, and 1.5T; Siemens Prisma, 3T). A human phantom study (planned publication in 2021) across the different scanners was performed with one healthy control and one stroke patient. The study protocol consisted of 3D-T1 weighted, axial T2, 3D-Fluid attenuated inversion recovery (FLAIR), diffusion-weighted imaging (DWI), and susceptibility-weighted imaging (SWI). Detailed description of the MRI protocol can be found in Supplementary Table 2. Causes of why patients declined participation in the MRI sub study were not recorded.

### Data Preparation

Cortical volumetric and thickness measurements were generated through cortical reconstruction and parcelation, and volumetric segmentation of the 3D-T1 scans. This was performed using the comprehensive recon-all process of Freesurfer 6.0.1 image analysis suite^1^ (Fischl, 2012). Cortical measurements were gathered into lobes (frontal, parietal, temporal, occipital, and cingulate) as suggested by Klein and Tourville (2012).

In preparation for WMH analysis, all MRI scans were reconstructed, denoised, deobliqued, and corrected for inhomogeneities. 3D-T1 scans were segmented into six tissue compartments (gray matter, white matter, cerebrospinal fluid (CSF), bone, soft tissue, and air/background), non-linearly warped into MNI space using the DARTEL algorithm, and smoothed with a 10 mm full-width half-maximum (FWHM) Gaussian kernel using voxel-based morphometry (VBM) in SPM 12, as previously described (Wright et al., 1995; Ashburner and Friston, 2000). The corresponding FLAIR images were then co-registered with the T1 into native subject space and normalized to MNI space using flow fields generated during the T1 processing.

### WMH Segmentation

White matter hyperintensities detection was performed using the fully automated and supervised FMRIB tool FSL BIANCA (Griffanti et al., 2016). BIANCA is based on a k-nearest neighbor algorithm and classifies the probability of WMHs based on the intensity and spatial features of the voxel. In order to create feature vectors for lesion classification, a training set of 36 manually segmented lesion masks were created, with the following number at each site: Oslo University Hospital = 10, Ullevål = 10, Baerum Hospital = 10, St. Olavs University Hospital = 10, Haukeland University Hospital = 5, and Ålesund Hospital = 1. BIANCA was run by separate training sets for each study location. For sites with 10 masks the training set consisted of solely local participants. For sites with <10 number of masks, the training set consisted of a mix of local participants and participants from an equivalent scanner. The intensity- and spatial-based lesion classification probability threshold was set to 0.7. An anatomical white matter mask from the T1 was used so as to exclude false positive hyperintensities occurring in regions outside the white matter. Volumetric lesion measurements of true WMH were calculated in mm^3^ and converted to milliliter (ml).

Due to a noticeable underestimation of WMH across multiple thresholds as well as a substantial number of false positive classifications of stroke lesions tissue as WMH in FSL BIANCA, manual editing of all BIANCA output was performed. This was done in accompany with a stroke mask based on the corresponding DWI-series in order to visualize the stroke lesion. A Wilcoxon-Mann-Whitney test was performed for calculation of the difference in volume between the automatic and the manually edited segmentations, revealing a significantly higher WMH volume after editing [mean (SD) of 24.7 (24.7) ml, as opposed to 20.4 (15.2) ml].

### Stroke Volume Extraction and Stroke Location

**FIGURE 1:**
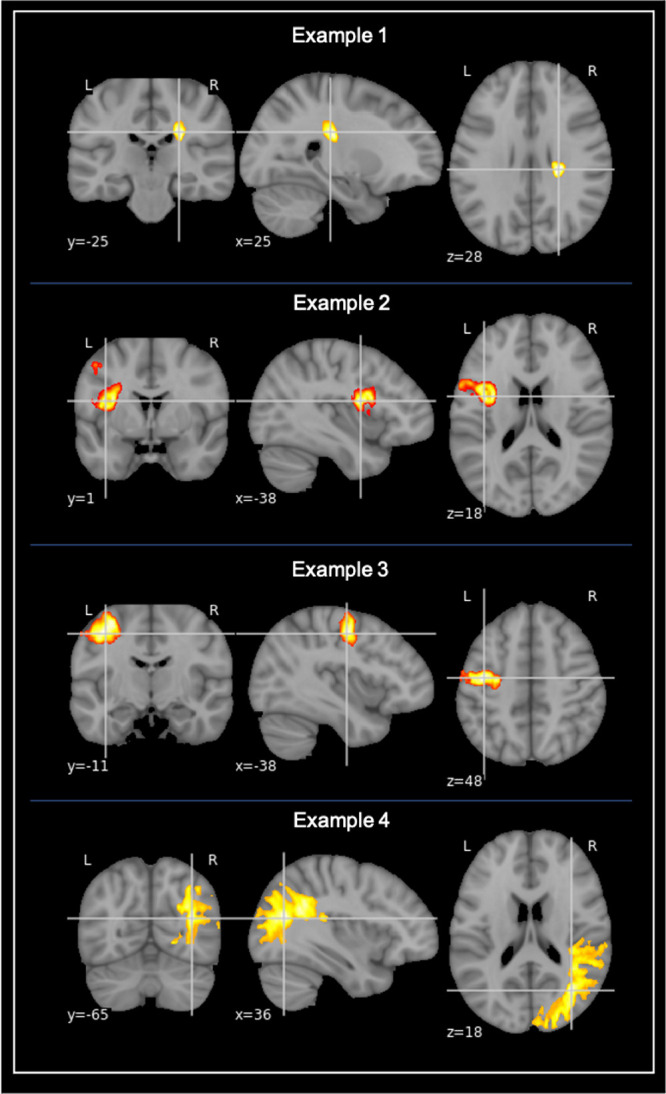
Examples of stroke lesion segmentation. Examples of voxel-based segmentation of stroke lesions in MNI space, as shown in the coronal, sagittal, and horizontal plane. Color map depicts a larger number of voxels and thus also a larger stroke increasing from red to yellow.

Stroke volume lesion masks were created for the patients who had visible diffusion restriction on DWI. The stroke lesion volume is equivalent to the ischemic core that is a proxy of the amount of irreversibly destroyed brain parenchyma, identified as diffusion restriction on the DWI sequence. The acute infarcts were semi-automatically labeled with the help of the ITK-Snap snake tool (v. 3.8.0) (Yushkevich et al., 2006) (see Figure 1). The masked stroke volume in mm^3^ was automatically measured by ITK-snap (v. 3.8.0) and converted to ml.

Stroke location was based on the lesion masks and determined using the Talairach lobe atlas (Lancaster et al., 1997, 2000). The labels “anterior lobe” and “posterior lobe” were then gathered into “Cerebellum,” and “medulla,” “midbrain,” and “pons” were gathered into “Brainstem.” If the stroke was labeled as “background,” the Harvard-Oxford structural atlas (Frazier et al., 2005; Desikan et al., 2006; Makris et al., 2006; Goldstein et al., 2007) was used instead. Stroke location was established by what lobe the highest percentage of the lesion was in. For participants with multiple lesions, the location of the largest lesion was used as stroke location.

### Clinical Characteristics

Demographic- and clinical data were collected at the time of the index stroke by study nurses and stroke physicians. Based on previous literature on factors that are either directly or indirectly associated with neurodegenerative-driven and/or vascular-driven post-stroke cognitive decline, the following data was included in our analysis: age, gender, education (years), BMI, smoking, and stroke severity measured using the “National Institute of Health Stroke Scale (NIHSS),” AF, pre-existing depression, the Charlson comorbidity index (CCI), and medications. The NIHSS scale ranges from 0 to 42, with higher scores indicating more severe strokes. AF was defined as patients having a (past or present) pathological ECG recording. Pre-existing depression was measured by self-report. The CCI involves weighing (from 1 to 6) of comorbidities, such that the higher the score, the more likely a mortality outcome. A CCI score of 3 or higher indicates high morbidity (Charlson et al., 2014). Medications were included as proxies for disease linked to risk of stroke and/or major NCD, with statins, antidiabetic medication, antihypertensive medication, anticoagulant medication, and antiplatelet medication being included. All medications were prescribed before admission to the hospital. List of medications and their corresponding condition can be found in Supplementary Table 1.

### Cognitive Assessment

The neuropsychological test battery included Trail making A and B (TMT A and B) (Reitan, 1958), ten word memory and recall test (CERAD) (Morris et al., 1988), the controlled oral word association test (COWAT) (Loonstra et al., 2001), the Montreal Cognitive Assessment (MoCA) (Nasreddine et al., 2005), the Ascertain Dementia 8-item informant questionnaire (AD-8) (Galvin et al., 2005), the Global Deterioration Scale (GDS) (Reisberg et al., 1982), Neuropsychiatric Inventory (NPI-Q) (Cumming et al., 2012), the Hospital Anxiety and Depression Scale (HADS) (Zigmond and Snaith, 1983), and the Cornell scale (Alexopoulos et al., 1988).

The diagnosis of NCD was based on the Diagnostic and Statistical Manual of Mental Disorders (DSM−5) criteria (American Psychiatric Association, 2013), which base diagnostic workups on both neuropsychological test scores and instrumental activities of daily living (I-ADL) (American Psychiatric Association, 2013). Patients scoring <–1.5 SD in at least one cognitive domain were defined as having post−stroke NCD.

Major NCD was defined as post−stroke NCD accompanied by dependency in I−ADL, whereas mild NCD was defined as post−stroke NCD without dependencies in any I−ADL, as described in previous work in the Nor-COAST study (Munthe-Kaas et al., 2020).

For the Support Vector Machine (SVM) (Vapnik, 1998) analysis in the current study, cognitive status at 3 months after the acute stroke was dichotomized and classified into major NCD versus normal/mild NCD (including both normal cognition and mild NCD).

Pre-stroke global cognition was measured using the GDS (Reisberg et al., 1982) and collected through interviews with relatives or caregivers. The scale ranges from 1 to 7, with the score of 3 representing mild NCD and 4 through 7 representing major NCD.

### Statistical Analysis

Means and standard deviations (SD) were calculated and normality was tested in the dependent variables. Mann-Whitney-*U* tests were run for the non-normally distributed continuous variables (WMH volume, stroke lesion volume, and right occipital cortical thickness). Student *t*-tests were run for the rest of the continuous variables with normal distribution (age, BMI, education, NIHSS stroke severity, CCI, and the remaining cortical thickness measures). Chi square tests were run for the categorical variables (gender, smoking, AF, pre-existing depression, and medication intake).

A support vector machine (Vapnik, 1998) is a popular machine learning algorithm used in order to perform pattern recognition and thus classify a best fitted model for prediction of an outcome. The algorithm finds a multidimensional plane that maximizes the margin between different class data points. Non-linear kernels can be applied to the algorithm, which allows for the use of non-planar, multidimensional surfaces to classify the pattern of the data. SVM classifiers were performed for the dichotomized classification of post-stroke neurocognitive outcome at 3 months – major NCD or normal/mild NCD. The radial basis function (RBF) kernel was implemented. The kernel’s width and SVM cost function were optimized through grid search, using the e1071 package (Dimitriadou et al., 2008) in R 3.6.0^2^. Variables were ranked based on the elements of a linear normal vector and those with lower weights were iteratively removed, creating a model with top *n* features (Guyon et al., 2002). The SVM algorithm was trained with all the clinical and imaging variables. A leave-one-out cross-validation (LOOCV) approach was used in order to predict each subject’s outcome and for model validation. The SVM was tuned to find the optimal hyperparameters for the model and the model’s predictive accuracy was measured by summing up the correct and incorrect classifications.

The following variables were used in the classifiers (in total 26 input variables): gender, age, education, BMI, smoking status, comorbidity, NIHSS stroke severity, AF, pre-existing depression, selected medication intake (statins, antidiabetics, antihypertensives, anticoagulants, and antiplatelet medication), lobar cortical thicknesses (frontal, parietal, temporal, occipital, and cingulate), stroke lesion volume, and WMH volume.

Next, we ran receiver-operating characteristic (ROC) models and obtained the area under the curve (AUC) for each classifier iteration. DeLong analysis DeLong et al. (1988) was used to compare the AUCs, with the goal to identify the model with the fewest features that performs just as well (not statistically different) from the best model.

In order to check if pre-stroke cognitive dysfunction was predictive of post-stroke progression to major NCD, a supplementary SVM model was additionally run, containing the same variables but with the addition of pre-stroke GDS as a measure of pre-stroke cognitive function (in total 27 input variables). We recursively ran three classifier models containing the same variables as the main model, now with the addition of previous infarction and previous intra-cerebral hemorrhage (in total 28 variables). The models were based on pre-stroke GDS status, such that model (1) included all participants; (2) excluded those with pre-stroke major NCD; and (3) excluded those with pre-stroke mild or major NCD. As above, DeLong analysis was used for statistical comparison of the AUCs. This was done to see the effect of having pre-stroke GDS available for prediction of NCD outcome.

## Results

### Study Population

From the full Nor-COAST population, 352 (43.2%) underwent a study-specific MRI scan that fulfilled all of the quality requirements for further analysis. Of these, 157 (44.6%) were female, the mean (SD) age was 72.8 (11.2) years and the mean (SD) stroke severity NIHSS score was 4 (4.9).

**FIGURE 2:**
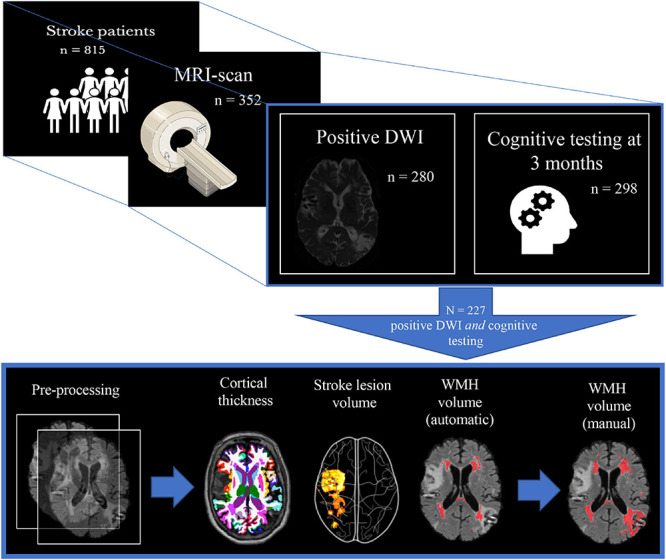
Study population selection with subsequent imaging processing. The figure depicts the inclusion process and subsequent imaging processing of the final study sample. From 815 participants, 352 underwent a study-specific MRI-scan, where 280 of these also had the stroke lesion visible on DWI, and 298 had cognitive testing at 3 months. This led to a final study population of 227 whereupon the following steps involved pre-processing of T1 + FLAIR MRI images: cortical thickness measures (FreeSurfer), stroke lesion volume (IKT SNAP), WMH volume (FSL BIANCA), and finally manual editing of the FSL BIANCA output. WMH, white matter hyperintensities.

A positive DWI was found in 280 (79.5%) and NCD status at 3 months was assessed in 298 (84.7%) of the MRI-sub study participants. Freesurfer anatomical segmentation was successfully performed in 331 (94%), stroke volume analysis in 280 (79.5%), and FSL BIANCA WMH analysis in 307 (87.2%). The FSL BIANCA output was manually edited for the participants who had NCD status at 3 months, successful Freesurfer segmentation, and stroke volume analysis; resulting in a final study sample of 227 (64.5%) (see Figure 2). The final study sample of 227 consisted of 99 (43.6%) females, with the mean age (SD) of 71.7 (11.3) years and a mean (SD) NIHSS stroke severity score of 3.8 (4.8).

### Baseline Characteristics

National Institute of Health Stroke Scale stroke severity scores generally fell within the “minor” category, with a mean (SD) NIHSS score of 3.8 (4.8). There was a high comorbidity burden, with a mean (SD) CCI level of 3.9 (1.9). Of the sample, 137 (60.4%) were either current- or ex-smokers, and the average BMI fell within the overweight category, with a mean (SD) of 26 (4.2). AF was present in 30 (13.2%) participants and only 9 (4%) reported pre-existing depression. Statins were prescribed in 73 (32.2%), 30 (13.2%) were on antidiabetic, and 112 (49.3%) on antihypertensive medications. Anticoagulants were prescribed in 20 (8.8%) of the participants, where 2 (10%) of these had previous cerebrovascular disease and 7 (35%) had previous coronary heart disease. Out of the 82 (36.1%) who were on antiplatelet medication prior to the stroke, 36 (43.9%) had previous cerebrovascular disease and 39 (47.6%) had previous coronary heart disease. At the 3-month follow-up, 63 (27.8%) were categorized as having mild NCD, whereas 62 (27.3%) had major NCD. Pre-stroke cognitive decline was low across the groups, with only those who had major NCD post stroke showing significant pre-stroke decline, with a mean (SD) GDS score of 2.2 (1.3), falling within the “very mild cognitive decline” category. As for stroke location, most strokes were found in the frontal and sub-cortical regions.

Compared to the normal/mild NCD group, the major NCD group were significantly older (77.4 vs. 69.6, *p* < 0.001), had fewer years of education (10.5 vs. 13, *p* < 0.001), more severe NIHSS stroke severity scale score (5.4 vs. 3.2, *p* < 0.001), and more comorbidities (CCI 4.8 vs. 3.6, *p* < 0.001). The major NCD group also had a significantly higher pre-stroke GDS score (*p* < 0.001), although not high enough for a diagnosis of NCD. Those in the normal/mild group were more likely to suffer from AF (*p* = 0.035), and to be on antidiabetics (*p* = 0.011), antihypertensives (*p* = 0.005), and antiplatelet medication (*p* < 0.001), but when looking at normal and mild NCD individually, the highest percentages of AF, antidiabetics, and antiplatelet medication were found in the major NCD group. No gender difference was found between the groups.

### MRI Markers

**FIGURE 3:**
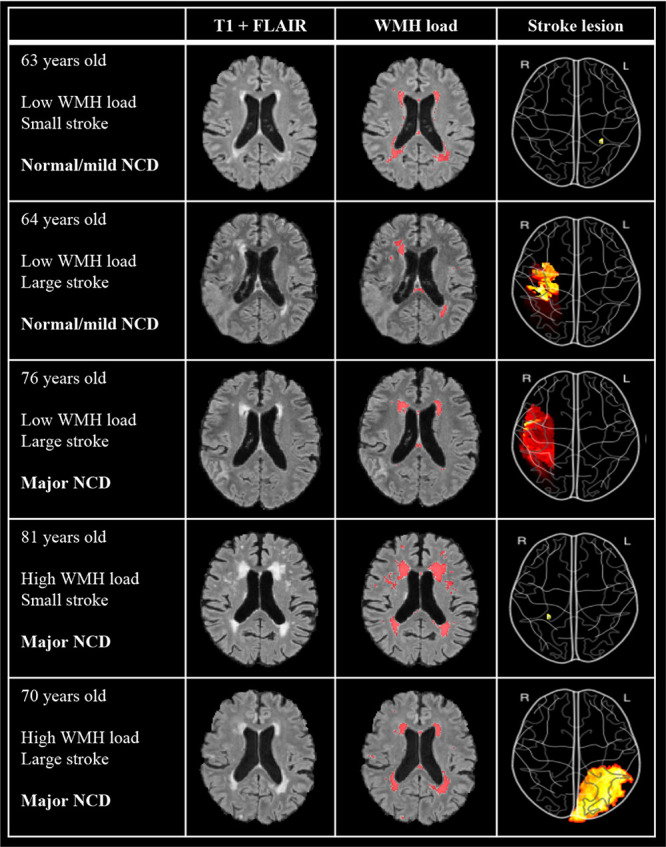
Examples of imaging findings of five participants with differing levels of WMH, stroke volumes, and NCD outcome. T1 + FLAIR image shows “untouched” co-registered image and WMH load shows the same co-registered image with annotated WMH from manually edited output from FSL BIANCA. Stroke lesion color map depicts volume projection, such that red indicates a larger stroke than yellow. Image is flipped so as to correspond to the hemisphere of MRI images. WMH, white matter hyperintensities; NCD, neurocognitive disorder.

As depicted in Table 1, WMH load with a mean (SD) of 38.7 (34.7) ml, and stroke lesions with a mean (SD) of 16.7 (28.2) ml, were largest in the major NCD group. WMH volume (*p* < 0.001) and stroke lesion volume (*p* = 0.019) were significantly larger in the major NCD group, whereas cortical thickness measures were found to be significantly smaller in the left hemisphere of the frontal (*p* < 0.001), parietal (*p* = 0.001), and temporal (*p* < 0.001) lobes. For examples of MRI findings, see Figure 3.

**TABLE 1:** Baseline characteristics and imaging findings by cognitive outcome group three months after stroke.

	**Normal/mild NCD**	**Major NCD**	**Total**	***p*-value**
*N*	165 (72.68%)	62 (27.31%)	227 (100%)	
**Demographic and clinical characteristics**				
Age (years)	69.56 ± 10.83	77.37 ± 10.41	71.69 ± 11.25	**<0.001**
Education (years)	13.01 ± 3.67	10.5 ± 3.21	12.33 ± 3.72	**<0.001**
BMI*	26.28 ± 4.26	25.32 ± 4.04	25.97 ± 4.19	0.127
NIHSS^1^	3.18 ± 3.52	5.42 ± 6.81	3.79 ± 4.75	**0.001**
Comorbidity	3.58 ± 1.85	4.79 ± 1.81	3.91 ± 1.91	**<0.001**
Pre-stroke GDS^2^	1.2 ± 0.44	2.16 ± 1.3	1.46 ± 0.88	**<0.001**
Female gender	69 (69.70%)	30 (30.30%)	99 (43.61%)	0.374
Smoking**				0.341
Never	67 (76.14%)	21 (23.86%)	38.77%)	
Smoker	39 (78%)	11 (22%)	50 (22.03%)	
Ex-smoker	58 (66.67%)	29 (33.33%)	87 (38.33%)	
Atrial fibrillation	17 (56.67%)	13 (43.33%)	30 (13.22%)	**0.035**
Pre-existing depression	7 (77.78%)	2 (22.22%)	9 (3.96%)	0.727
Statins	47 (64.39%)	26 (35.62%)	73 (32.16%)	0.053
Antidiabetic med.	16 (53.34%)	14 (46.67%)	30 (13.22%)	**0.011**
Antihypertensive med.	72 (64.29%)	40 (35.71%)	112 (49.34%)	**0.005**
Anticoagulant med.	13 (65%)	7 (35%)	20 (8.81%)	0.419
Antiplatelet med.	48 (58.54%)	34 (41.46%)	82 (36.12%)	**<0.001**
**MRI findings**				
WMH (ml)	21.95 ± 19.99	38.68 ± 34.68	26.52 ± 25.89	**<0.001**
Stroke lesion (ml)	5.75 ± 9.54	16.71 ± 28.23	8.74 ± 17.47	**0.019**
Frontal th. (left) (mm)*	2.34 ± 0.13	2.24 ± 0.14	2.32 ± 0.14	**<0.001**
Frontal th. (right) (mm)*	2.30 ± 0.13	2.30 ± 0.17	2.30 ± 0.14	0.870
Parietal th. (left) (mm)*	2.13 ± 0.14	2.06 ± 0.14	2.11 ± 0.15	**0.001**
Parietal th. (right) (mm)*	2.11 ± 0.15	2.11 ± 0.18	2.11 ± 0.16	0.919
Temporal th (left) (mm)*	2.70 ± 0.17	2.54 ± 16	2.67 ± 0.18	**<0.001**
Temporal th. (right) (mm)*	2.69 ± 0.16	2.66 ± 0.19	2.68 ± 0.17	0.204
Occipital th. (left) (mm)*	1.85 ± 0.12	1.82 ± 0.14	1.84 ± 0.12	0.204
Occipital th. (right) (mm)*	1.89 ± 0.17	1.91 ± 0.22	1.89 ± 0.18	0.585
Cingulate th. (left) (mm)*	2.49 ± 0.19	2.44 ± 0.23	2.48 ± 0.20	0.066
Cingulate th. (right) (mm)*	2.47 ± 0.19	2.49 ± 0.20	2.48 ± 0.20	0.591
***Stroke location***				0.171
Frontal	50 (73.53%)	18 (26.47%)	68 (29.96%)	
Temporal	5 (45.45%)	6 (54.55%)	11 (4.85%)	
Occipital	15 (60.00%)	10 (40.00%)	25 (11.01%)	
Parietal	20 (71.43%)	8 (28.57%)	28 (12.33%)	
Sub-cortical	35 (74.47%)	12 (25.53%)	47 (20.70%)	
Limbic	5 (71.43%)	2 (28.57%)	7 (3.08%)	
Brainstem	15 (78.95%)	4 (21.05%)	19 (8.37%)	
Cerebellum	20 (90.91%)	2 (9.09%)	22 (9.69%)	

*P-values testing the null hypothesis that there is a difference between normal/mild NCD vs. major NCD groups. Significant p-values indicated in bold.*

Percentages given in the two NCD-groups are of the total of the factor, whereas the percentage within the total is of the total *N*. Stroke location of principal stroke, using the Talairach structural atlas. Th., cortical thickness.

*Imputed values in 12–20 subjects.

**Missing data in two subjects.

*^1^NIHSS, National Institute of Health Stroke Scale.*

*^2^GDS, Global Deterioration Scale.*

### SVM Results

The best SVM model selected 19 of the 26 features and achieved an AUC of 0.876. The DeLong analysis revealed that the AUC of the model with the eight top features (AUC = 0.850) was not significantly different from the model with 19 features. The supplementary SVM model including pre-stroke GDS achieved an AUC of 0.878 and was driven by 9 of the 27 features (pre-stroke GDS in addition to the main 26 features) (see Table 2).

**TABLE 2:** Factors in training of support vector machine (SVM) and the three output models.

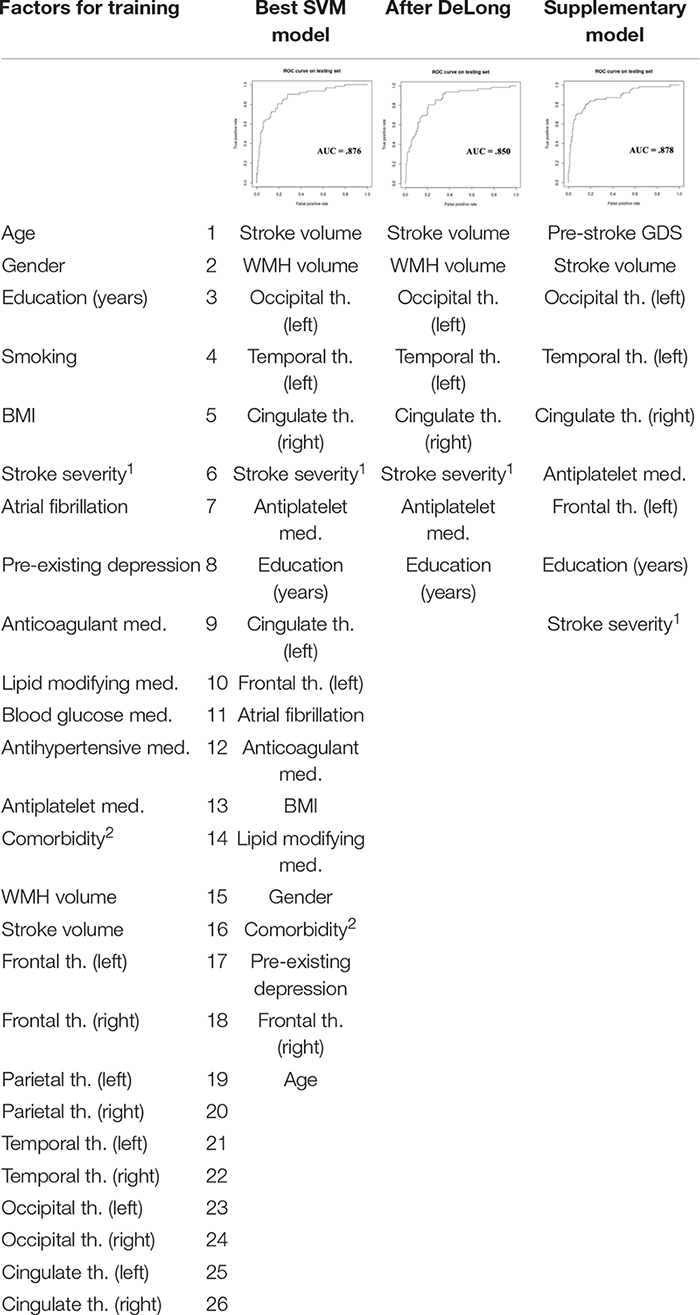

*ROC curves and AUC per SVM output models – before and after DeLong analysis, and the supplementary model also containing pre-stroke GDS. Factors listed in descending order according to weight (except in the “factors for training”-column, which is listed unordered).*

*th., cortical thickness.*

*^1^NIHSS, National Institute of Health Stroke Scale.*

*^2^Charlson Comorbidity Index.*

For model comparison between the models including pre-stroke GDS or not, pre-stroke GDS correlation with WMH volume was measured, showing significant correlation in both the mild (*r* = 0.3, *p* = 0.03) and the major (*r* = 0.5, *p* < 0.001) NCD groups.

The classifier model based on pre-stroke GDS containing all of the participants selected 18 of the 28 features and achieved an AUC of 0.874. The model including those with normal cognition or only mild NCD prior to the stroke selected 12 of the 28 features and achieved an AUC of 0.855. The model including only participants with normal cognition before the stroke selected 9 of the 28 features and achieved an AUC of 0.802. For feature weighting, please see Supplementary Table 3.

To test whether the 18-feature model performed just as well when individuals with pre-stroke major cognitive impairment were excluded, we ran the 18-feature model in a sample consisting of only participants with normal cognition and mild NCD. This model achieved an AUC of 0.834. The AUC of this model was not significantly different from the 12-feature classifier result in the same population (*Z* = 1.108, *p* = 0.268).

## Discussion

The aim of the current study was to identify clinical and imaging factors that can predict the development of rapid onset of major NCD after a stroke. We hypothesized that the prediction would mainly be driven by neurodegenerative factors. Overall, the participants in the major NCD group showed significantly larger stroke and WMH volumes, and smaller cortical thicknesses in the left hemisphere. We also found that the best model for NCD outcome prediction included both neurodegenerative and vascular markers, with the top eight factors being stroke lesion volume, WMH volume, left hemisphere occipital and temporal thickness, right hemisphere cingulate thickness, NIHSS stroke severity, antiplatelet medicine intake, and education.

In line with previous literature, stroke lesion volumes were found to be higher in the major NCD group, with a mean volume of 16.7 vs. 5.8 ml in the normal/mild NCD group. Due to its potential to destroy or compromise tissue vital for cognitive function, stroke lesion volume, as well as WMH load, has been found to be an important independent predictor of post-stroke cognitive deficits (Pendlebury, 2012; Puy et al., 2018; Georgakis et al., 2019), at least for volumes larger than 5 ml (Molad et al., 2019).

White matter hyperintensities volumes were also generally higher in the major NCD group. WMHs are commonly presumed to be of vascular origin and may be due to a compromised blood brain barrier, hypertension, and the degeneration of axons and myelin. It is associated with cognitive decline and is found in a multitude of neurological diseases, but is also common in clinically healthy aging (Prins and Scheltens, 2015; d’Arbeloff et al., 2019). No absolute gold standard is set for what amount of WMH load is outside of the healthy realm, but studies have found WMH loads of up to 32 ml in clinically healthy adults (Raz et al., 2012; De Marco et al., 2017). The current study found mean WMH loads beyond this cut-off only in the major NCD group, but all groups showed max loads well above this. This finding is probably due to our cohort showing an array of vascular risk factors that are found associated with WMHs. Also, the normal/mild NCD group include patients with mild NCD, which is also associated with having more WMH (Pendlebury, 2012). This variance may, however, merely come down to an inconsistency across projects in how WMH is estimated and thus also the volume reported, pointing at a need for a methodological gold standard in the field (Kuijf et al., 2019).

Cortical thickness in the temporal and occipital left hemisphere were found to be less thick in the major NCD group. A thinner cortex in general has previously been found to be an independent predictor of cognitive impairment in both healthy controls and dementia cohorts (Apostolova et al., 2007), but not an independent predictor in stroke cohorts (Dickie et al., 2020). A thinner cortex in temporal regions rather than in more superior lobes have also been found to be associated with a higher WHM load (Dickie et al., 2020). Other studies suggest that, also in healthy adults, the association between WMHs and cognitive impairment is mediated by a reduced cortical thickness (Zi et al., 2014). It therefore seems that there is an intricate interaction between WMH, cortical thickness, and cognitive decline at play.

The current study found no significant difference between the groups for cingulate cortex thickness, although it was a main predictor in the SVM model. Atrophy and related functional connectivity damages in the cingulate cortex has been found to be associated with cognitive decline (Belkhiria et al., 2019; Cera et al., 2019). The cingulate cortex is a network hub within the brain that is linked with multiple networks and is thus associated with many functions related to cognition, mood, and behavior (Larivière et al., 2018; Rolls, 2019). General cortical atrophy is common with normal aging, but predominantly in the right hemisphere and the frontal regions of the brain (Hurtz et al., 2014). Cingulate atrophy can also be found with healthy aging, but is more commonly associated with neurodegenerative disease, such as AD (Touroutoglou and Dickerson, 2019). Delayed atrophy of the posterior cingulate cortex has also been found after stroke (>6 months), where the severity of the volumetric change was associated with apathy (Matsuoka et al., 2015; Haque et al., 2019), a common neuropsychiatric feature seen with cognitive decline and dementia (van Dalen et al., 2018). This finding could, however, also be explained by underlying vascular disease (Wouts et al., 2020), again highlighting the complex interplay between vascular changes and neurodegeneration and also the timing of onset for cognitive decline. The review by Mok et al. (2017) was concluded with the proposition that early-onset post-stroke NCD is linked primarily to stroke lesion characteristics and brain resilience, whereas delayed-onset is linked to small vessel disease. The authors’ idea was that early onset cognitive decline after a stroke happens either if the stroke is mild but the brain is not resilient enough to recover from the insult, or if the stroke is too large or hits strategically, such that even a high resilience brain is left defenseless. Late-onset, they continued, appears, unless there is a recurrent stroke, primarily due to coexistent cerebrovascular disease. These patients are more at risk of having small subcortical infarcts rather than cortical infarcts and thereby less likely to develop early onset NCD. The findings of the current study do, however, not support the proposition put forth by Mok et al., as we found that early onset is linked to both neurodegenerative and vascular changes, with in fact *vascular* changes being the more important factors of the two. This divergence highlights the complexity and the need for further research of post-stroke NCD.

Most of the differences in cortical thickness between the groups were found in the left hemisphere (all regions except occipital and cingulate cortex). This is not surprising, as left hemisphere strokes are generally associated with vascular dementia and worse outcome (Mijajlović et al., 2017). Another expected finding was that there were more severe strokes in the major NCD group. More severe strokes have been found to be an important predictor of cognitive decline (Ferreira et al., 2015) and dementia (Pendlebury and Rothwell, 2009). The major NCD group also had a lower level of education than did the rest. This is in line with previous reports, as education has been found to serve as a protective factor such that higher education is associated with a lower risk of suffering from post-stroke NCD (Pendlebury and Rothwell, 2009; Lövdén et al., 2020).

The participants in the major NCD group were more likely to be on antiplatelet medication prior to the stroke than did the other two groups. Antiplatelet medicine is a common antithrombotic drug and secondary preventive treatment. Antiplatelets are often given to patients at risk of stroke, either due to having some of the risk factors for stroke or having already suffered a transient ischemic attack (TIA) or stroke – all risks also associated with cognitive decline (Pendlebury and Rothwell, 2009; Kernan et al., 2014). Of those on antiplatelets, 43.9% of our participants had previous cerebrovascular disease and 47.6% previous coronary heart disease, indicating vascular disease and thus also a risk of both stroke and cognitive decline.

No significant gender difference was found between the NCD groups, although gender was one of the 19 factors in the prediction model. Although a significant part of the model, its prediction weight left it far down on the list. It is generally found that women are at higher risk for post-stroke dementia, mainly due to a longer life-expectancy (Carcel et al., 2020). A study using the same data as the current study (Schellhorn et al., 2021) proposed that the absence of gender difference may come down to a counterbalancing effect happening in this cohort, as it was found that although the women were older, they were also more likely to have fewer lacunes, pathological MTA scores and fewer pathological imaging finding.

Pre-stroke cognitive status was not included in the current main analysis, as we aimed to create a prediction model with factors typically available in a clinical setting. Pre-stroke cognitive status is an important predictor of post-stroke cognitive status (Pendlebury, 2012), but pre-stroke GDS may not be routinely obtained in a clinical setting. As a supplementary test, we ran a model including pre-stroke GDS. This led to a very similar AUC as the main model, but it resulted in fewer factors and WMH volume completely disappeared from the best model. Next, the additional sensitivity analysis revealed that the model containing all of the participants was statistically not significantly different from the model excluding those with pre-stroke major NCD. Just as with the supplementary SVM model containing pre-stroke GDS as a factor, the biggest difference was that WMH volume disappeared from the model. This confirms the sensitivity of the main model, but also indicates that pre-stroke GDS can potentially be explained by WMHs and that one may be able to use WMH volume as a surrogate marker of pre-stroke GDS, if it is not available. This finding is backed up by Puy et al. (2018), who also did not find WMH load to be an independent predictor of pre-stroke cognitive decline. This may be due to a strong relationship between WMH load and pre-stroke GDS, and that pre-stroke GDS is a better predictor, thus leaving WMH volume superfluous. As WMH load is strongly associated with cognitive decline even when there is no stroke, this finding is not too surprising.

This study has several strengths and weaknesses that must be acknowledged. First off, this study involves a large dataset that includes thorough examination of clinical, neuropsychological, and imaging factors at time of index stroke and neuropsychological factors at a 3-month follow-up. Secondly, the prediction model is based on what is typically available in a clinical setting at an acute stroke event, thus focusing in on clinical application. Lastly, due to the less than desirable automated segmentation of WMHs, the study contains a large set of manually edited WMH masks that have been through a thorough quality control.

This study is limited by the loss of participants, with a final sample of only 64.5% of those who underwent an MRI, and 27.9% of the full Nor-COAST participant pool. This was mostly due to lack of cognitive testing and participation in the MRI sub study, respectively. Also, the current study mostly includes patients with mild to moderate strokes, which makes the results less generalizable to patients who suffer severe strokes. Future studies could prevent this through basing the study on standard clinical protocols, so as to remove the need for a second (study-specific) scan. Nevertheless, an investigation on the generalizability of Nor-COAST found that although more severe strokes are excluded, the findings are indeed comparable to the Norwegian Stroke Registry (Kuvås et al., 2020).

## Conclusion

The development of rapid onset major NCD after stroke is possible to predict with an 87.6% accuracy using a mix of clinical and imaging factors. The prediction of rapid onset major NCD seems dependent not on neurodegenerative factors alone, but also on vascular factors, as well as aspects of the stroke itself, such as stroke lesion size. In contrast to previous literature, we also found that vascular changes are more important than the neurodegenerative brain changes. Although possible to predict with relatively high accuracy, our findings indicate that the development of rapid onset post-stroke NCD may be more complex than earlier suggested.

## Data Availability Statement

The raw data supporting the conclusions of this article will be made available by the authors, without undue reservation.

## Ethics Statement

The studies involving human participants were reviewed and approved by REK Nord. The patients/participants provided their written informed consent to participate in this study.

## Author Contributions

EA: software, formal analysis, data curation, writing – original draft, writing – review and editing, visualization, and project administration. TS: software, formal analysis, data curation, writing – review and editing, and visualization. ES, AS, and PL: software and writing – review and editing. DS: software. LA: methodology, writing – review and editing, and supervision. IS: writing – review and editing and funding acquisition. MB: conceptualization, methodology, writing – review and editing, supervision, project administration, and funding acquisition. All authors contributed to the article and approved the submitted version.

## Conflict of Interest

The authors declare that the research was conducted in the absence of any commercial or financial relationships that could be construed as a potential conflict of interest.

## Publisher’s Note

All claims expressed in this article are solely those of the authors and do not necessarily represent those of their affiliated organizations, or those of the publisher, the editors and the reviewers. Any product that may be evaluated in this article, or claim that may be made by its manufacturer, is not guaranteed or endorsed by the publisher.

## Footnotes

1
http://surfer.nmr.mgh.harvard.edu/

2
http://cran.r-project.org

## References

[B1] Alexopoulos G. S. Abrams R. C. Young R. C. Shamoian C. A. (1988). Cornell scale for depression in dementia. *Biol. Psychiatry* 23 271–284. 10.1016/0006-3223(88)90038-8 3337862

[B2] American Psychiatric Association A. P. (2013). *Diagnostic and Statistical Manual of Mental Disorders*, 5 th Edn. Virginia, VA: The American Psychiatric Association.

[B3] Apostolova L. G. Steiner C. A. Akopyan G. G. Dutton R. A. Hayashi K. M. Toga A. W. (2007). Three-dimensional gray matter atrophy mapping in mild cognitive impairment and mild Alzheimer disease. *Arch. Neurol.* 64 1489–1495. 10.1001/archneur.64.10.1489 17923632 PMC3197839

[B4] Ashburner J. Friston K. J. (2000). Voxel-based morphometry–the methods. *Neuroimage* 11 805–821. 10.1006/nimg.2000.0582 10860804

[B5] Belkhiria C. Vergara R. C. San Martín S. Leiva A. Marcenaro B. Martinez M. (2019). Cingulate cortex atrophy is associated with hearing loss in presbycusis with cochlear amplifier dysfunction. *Front. Aging Neurosci.* 11:97. 10.3389/fnagi.2019.00097 31080411 PMC6497796

[B6] Carcel C. Woodward M. Wang X. Bushnell C. Sandset E. C. (2020). Sex matters in stroke: a review of recent evidence on the differences between women and men. *Front. Neuroendocrinol.* 59:100870. 10.1016/j.yfrne.2020.100870 32882229

[B7] Casolla B. Caparros F. Cordonnier C. Bombois S. Hénon H. Bordet R. (2019). Biological and imaging predictors of cognitive impairment after stroke: a systematic review. *J. Neurol.* 266 2593–2604. 10.1007/s00415-018-9089-z 30350168

[B8] Cera N. Esposito R. Cieri F. Tartaro A. (2019). Altered cingulate cortex functional connectivity in normal aging and mild cognitive impairment. *Front. Neurosci.* 13:857. 10.3389/fnins.2019.00857 31572106 PMC6753224

[B9] Charlson M. Wells M. T. Ullman R. King F. Shmukler C. (2014). The Charlson comorbidity index can be used prospectively to identify patients who will incur high future costs. *PLoS One* 9:e112479. 10.1371/journal.pone.0112479 25469987 PMC4254512

[B10] Cumming T. B. Tyedin K. Churilov L. Morris M. E. Bernhardt J. (2012). The effect of physical activity on cognitive function after stroke: a systematic review. *Int. Psychogeriatr.* 24 557–567. 10.1017/s1041610211001980 21996131

[B11] d’Arbeloff T. Elliott M. L. Knodt A. R. Melzer T. R. Keenan R. Ireland D. (2019). White matter hyperintensities are common in midlife and already associated with cognitive decline. *Brain Commun.* 1:fcz041. 10.1093/braincomms/fcz041 31894208 PMC6928390

[B12] DeLong E. R. DeLong D. M. Clarke-Pearson D. L. (1988). Comparing the areas under two or more correlated receiver operating characteristic curves: a nonparametric approach. *Biometrics* 44 837–845. 10.2307/2531595 3203132

[B13] Desikan R. S. Ségonne F. Fischl B. Quinn B. T. Dickerson B. C. Blacker D. (2006). An automated labeling system for subdividing the human cerebral cortex on MRI scans into gyral based regions of interest. *Neuroimage* 31 968–980. 10.1016/j.neuroimage.2006.01.021 16530430

[B14] De Marco M. Manca R. Mitolo M. Venneri A. (2017). White matter hyperintensity load modulates brain morphometry and brain connectivity in healthy adults: a neuroplastic mechanism? *Neural Plast* 2017:4050536. 10.1155/2017/4050536 28845309 PMC5560090

[B15] Dickie D. A. Gardner K. Wagener A. Wyss A. Arba F. Wardlaw J. M. (2020). Cortical thickness, white matter hyperintensities, and cognition after stroke. *Int. J. Stroke* 15 46–54. 10.1177/1747493019851291 31088224

[B16] Dimitriadou E. Hornik K. Leisch F. Meyer D. Weingessel A. (2008). *Misc functions of the Department of Statistics (e1071). TU Wien.* R Package Version 1 5–24.

[B17] Ferreira M. G. R. Moro C. H. C. Franco S. C. (2015). Cognitive performance after ischaemic stroke. *Dement. Neuropsychol.* 9 165–175. 10.1590/1980-57642015DN92000011 29213958 PMC5619355

[B18] Fischl B. (2012). FreeSurfer. *Neuroimage* 62 774–781. 10.1016/j.neuroimage.2012.01.021 22248573 PMC3685476

[B19] Frazier J. A. Chiu S. Breeze J. L. Makris N. Lange N. Kennedy D. N. (2005). Structural brain magnetic resonance imaging of limbic and thalamic volumes in pediatric bipolar disorder. *Am. J. Psychiatry* 162 1256–1265. 10.1176/appi.ajp.162.7.1256 15994707

[B20] Galvin J. E. Roe C. M. Powlishta K. K. Coats M. A. Muich S. J. Grant E. (2005). The AD8: a brief informant interview to detect dementia. *Neurology* 65 559–564. 10.1212/01.wnl.0000172958.95282.2a 16116116

[B21] Georgakis M. K. Duering M. Wardlaw J. M. Dichgans M. (2019). WMH and long-term outcomes in ischemic stroke: a systematic review and meta-analysis. *Neurology* 92 1298–1308.e. 10.1212/wnl.0000000000007142 30770431

[B22] Goldstein J. M. Seidman L. J. Makris N. Ahern T. O’Brien L. M. Caviness V. S. Jr. (2007). Hypothalamic abnormalities in schizophrenia: sex effects and genetic vulnerability. *Biol. Psychiatry* 61 935–945. 10.1016/j.biopsych.2006.06.027 17046727

[B23] Griffanti L. Zamboni G. Khan A. Li L. Bonifacio G. Sundaresan V. (2016). BIANCA (Brain Intensity AbNormality Classification Algorithm): a new tool for automated segmentation of white matter hyperintensities. *Neuroimage* 141 191–205. 10.1016/j.neuroimage.2016.07.018 27402600 PMC5035138

[B24] Guyon I. Weston J. Barnhill S. Vapnik V. (2002). Gene selection for cancer classification using support vector machines. *Mach. Learn.* 46 389–422. 10.1023/A:1012487302797

[B25] Haque M. E. Gabr R. E. Hasan K. M. George S. Arevalo O. D. Zha A. (2019). Ongoing secondary degeneration of the limbic system in patients with ischemic stroke: a longitudinal MRI study. *Front. Neurol.* 10:154. 10.3389/fneur.2019.00154 30890995 PMC6411642

[B26] Hurtz S. Woo E. Kebets V. Green A. E. Zoumalan C. Wang B. (2014). Age effects on cortical thickness in cognitively normal elderly individuals. *Dement. Geriatr. Cogn. Dis. Extra* 4 221–227. 10.1159/000362872 25177330 PMC4132234

[B27] Ihle-Hansen H. Thommessen B. Wyller T. B. Engedal K. Øksengård A. R. Stenset V. (2011). Incidence and subtypes of MCI and dementia 1 year after first-ever stroke in patients without pre-existing cognitive impairment. *Dement. Geriatr. Cogn. Disord.* 32 401–407. 10.1159/000335361 22311341

[B28] Kernan W. N. Ovbiagele B. Black H. R. Bravata D. M. Chimowitz M. I. Ezekowitz M. D. (2014). Guidelines for the prevention of stroke in patients with stroke and transient ischemic attack. *Stroke* 45 2160–2236. 10.1161/STR.0000000000000024 24788967

[B29] Klein A. Tourville J. (2012). 101 labeled brain images and a consistent human cortical labeling protocol. *Front. Neurosci.* 6:171. 10.3389/fnins.2012.00171 23227001 PMC3514540

[B30] Kuijf H. Biesbroek M. de Bresser J. Heinen R. Andermatt S. Bento M. (2019). Standardized assessment of automatic segmentation of white matter hyperintensities; results of the WMH segmentation challenge. *IEEE Trans. Med. Imaging* 38 2556–2568. 10.1109/TMI.2019.2905770 30908194 PMC7590957

[B31] Kuvås K. R. Saltvedt I. Aam S. Thingstad P. Ellekjær H. Askim T. (2020). The risk of selection bias in a clinical multi-center cohort study. Results from the norwegian cognitive impairment after stroke (Nor-COAST) study. *Clin. Epidemiol.* 12 1327–1336. 10.2147/clep.S276631 33293871 PMC7718873

[B32] Lancaster J. L. Rainey L. H. Summerlin J. L. Freitas C. S. Fox P. T. Evans A. C. (1997). Automated labeling of the human brain: a preliminary report on the development and evaluation of a forward-transform method. *Hum. Brain Mapp.* 5 238–242. 10.1002/(sici)1097-019319975:4<238::Aid-hbm6<3.0.Co;2-4 20408222 PMC2860189

[B33] Lancaster J. L. Woldorff M. G. Parsons L. M. Liotti M. Freitas C. S. Rainey L. (2000). Automated Talairach atlas labels for functional brain mapping. *Hum. Brain Mapp.* 10 120–131. 10.1002/1097-0193(200007)10:3<120::aid-hbm30<3.0.co;2-8 10912591 PMC6871915

[B34] Larivière S. Ward N. S. Boudrias M. H. (2018). Disrupted functional network integrity and flexibility after stroke: relation to motor impairments. *Neuroimage Clin.* 19 883–891. 10.1016/j.nicl.2018.06.010 29946512 PMC6008503

[B35] Loonstra A. S. Tarlow A. R. Sellers A. H. (2001). COWAT metanorms across age, education, and gender. *Appl. Neuropsychol.* 8 161–166. 10.1207/s15324826an0803_5 11686651

[B36] Lövdén M. Fratiglioni L. Glymour M. M. Lindenberger U. Tucker-Drob E. M. (2020). Education and cognitive functioning across the life span. *Psychol. Sci. Public Interest* 21 6–41. 10.1177/1529100620920576 32772803 PMC7425377

[B37] Makris N. Goldstein J. M. Kennedy D. Hodge S. M. Caviness V. S. Faraone S. V. (2006). Decreased volume of left and total anterior insular lobule in schizophrenia. *Schizophr. Res.* 83 155–171. 10.1016/j.schres.2005.11.020 16448806

[B38] Matsuoka K. Yasuno F. Taguchi A. Yamamoto A. Kajimoto K. Kazui H. (2015). Delayed atrophy in posterior cingulate cortex and apathy after stroke. *Int. J. Geriatr. Psychiatry* 30 566–572. 10.1002/gps.4185 25092799

[B39] Mellon L. Brewer L. Hall P. Horgan F. Williams D. Hickey A. (2015). Cognitive impairment six months after ischaemic stroke: a profile from the ASPIRE-S study. *BMC Neurol.* 15:31. 10.1186/s12883-015-0288-2 25879880 PMC4359388

[B40] Mijajlović M. D. Pavlović A. Brainin M. Heiss W.-D. Quinn T. J. Ihle-Hansen H. B. (2017). Post-stroke dementia-a comprehensive review. *BMC Med.* 15:11. 10.1186/s12916-017-0779-7 28095900 PMC5241961

[B41] Mok V. C. Lam B. Y. Wong A. Ko H. Markus H. S. Wong L. K. (2017). Early-onset and delayed-onset poststroke dementia-revisiting the mechanisms. *Nat. Rev. Neurol.* 13 148–159. 10.1038/nrneurol.2017.16 28211452

[B42] Molad J. Hallevi H. Korczyn A. D. Kliper E. Auriel E. Bornstein N. M. (2019). Vascular and neurodegenerative markers for the prediction of post-stroke cognitive impairment: results from the TABASCO study. *J. Alzheimers Dis.* 70 889–898. 10.3233/jad-190339 31282420

[B43] Morris J. C. Mohs R. C. Rogers H. Fillenbaum G. Heyman A. (1988). Consortium to establish a registry for Alzheimer’s disease (CERAD) clinical and neuropsychological assessment of Alzheimer’s disease. *Psychopharmacol. Bull.* 24 641–652. 3249766

[B44] Munthe-Kaas R. Aam S. Ihle-Hansen H. Lydersen S. Knapskog A. B. Wyller T. B. (2020). Impact of different methods defining post-stroke neurocognitive disorder: The Nor-COAST study. *Alzheimers Dement (N. Y.)* 6:e12000. 10.1002/trc2.12000 32211505 PMC7085256

[B45] Murray C. J. Vos T. Lozano R. Naghavi M. Flaxman A. D. Michaud C. (2012). Disability-adjusted life years (DALYs) for 291 diseases and injuries in 21 regions, 1990-2010: a systematic analysis for the Global Burden of Disease Study 2010. *Lancet* 380 2197–2223. 10.1016/s0140-6736(12)61689-4 23245608

[B46] Nasreddine Z. S. Phillips N. A. Bédirian V. Charbonneau S. Whitehead V. Collin I. (2005). The montreal cognitive assessment. MoCA: a brief screening tool for mild cognitive impairment. *J. Am. Geriatr. Soc.* 53 695–699. 10.1111/j.1532-5415.2005.53221.x 15817019

[B47] Pendlebury S. T. (2009). Stroke-related dementia: rates, risk factors and implications for future research. *Maturitas* 64 165–171. 10.1016/j.maturitas.2009.09.010 19818568

[B48] Pendlebury S. T. (2012). Dementia in patients hospitalized with stroke: rates, time course, and clinico-pathologic factors. *Int. J. Stroke* 7 570–581. 10.1111/j.1747-4949.2012.00837.x 22781124

[B49] Pendlebury S. T. Rothwell P. M. (2009). Prevalence, incidence, and factors associated with pre-stroke and post-stroke dementia: a systematic review and meta-analysis. *Lancet. Neurol.* 8 1006–1018. 10.1016/S1474-4422(09)70236-4 19782001

[B50] Pendlebury S. T. Rothwell P. M. (2019). Incidence and prevalence of dementia associated with transient ischaemic attack and stroke: analysis of the population-based Oxford Vascular Study. *Lancet Neurol.* 18 248–258. 10.1016/s1474-4422(18)30442-3 30784556 PMC6390174

[B51] Prins N. D. Scheltens P. (2015). White matter hyperintensities, cognitive impairment and dementia: an update. *Nat. Rev. Neurol.* 11 157–165. 10.1038/nrneurol.2015.10 25686760

[B52] Puy L. Barbay M. Roussel M. Canaple S. Lamy C. Arnoux A. (2018). Neuroimaging determinants of poststroke cognitive performance. *Stroke* 49 2666–2673. 10.1161/STROKEAHA.118.021981 30355190

[B53] Raz N. Yang Y. Dahle C. L. Land S. (2012). Volume of white matter hyperintensities in healthy adults: contribution of age, vascular risk factors, and inflammation-related genetic variants. *Biochimi. Biophys. Acta* 1822 361–369. 10.1016/j.bbadis.2011.08.007 21889590 PMC3245802

[B54] Reisberg B. Ferris S. H. de Leon M. J. Crook T. (1982). The Global Deterioration Scale for assessment of primary degenerative dementia. *Am. J. Psychiatry* 139 1136–1139. 10.1176/ajp.139.9.1136 7114305

[B55] Reitan R. M. (1958). Validity of the trail making test as an indicator of organic brain damage. *Percept. Mot. Skills* 8 271–276. 10.2466/pms.1958.8.3.271

[B56] Rolls E. T. (2019). The cingulate cortex and limbic systems for emotion, action, and memory. *Brain Struct. Funct.* 224 3001–3018. 10.1007/s00429-019-01945-2 31451898 PMC6875144

[B57] Sachdev P. S. Blacker D. Blazer D. G. Ganguli M. Jeste D. V. Paulsen J. S. (2014). Classifying neurocognitive disorders: the DSM-5 approach. *Nat. Rev. Neurol.* 10 634–642. 10.1038/nrneurol.2014.181 25266297

[B58] Schellhorn T. Zucknick M. Askim T. Munthe-Kaas R. Ihle-Hansen H. Seljeseth Y. M. (2021). Pre-stroke cognitive impairment is associated with vascular imaging pathology: a prospective observational study. *BMC Geriatr.* 21:362. 10.1186/s12877-021-02327-2 34126944 PMC8201706

[B59] Schneider J. A. Arvanitakis Z. Leurgans S. E. Bennett D. A. (2009). The neuropathology of probable Alzheimer disease and mild cognitive impairment. *Ann. Neurol.* 66 200–208. 10.1002/ana.21706 19743450 PMC2812870

[B60] Thiel A. Cechetto D. F. Heiss W. D. Hachinski V. Whitehead S. N. (2014). Amyloid burden, neuroinflammation, and links to cognitive decline after ischemic stroke. *Stroke* 45 2825–2829. 10.1161/strokeaha.114.004285 25005439

[B61] Thingstad P. Askim T. Beyer M. K. Bråthen G. Ellekjær H. Ihle-Hansen H. (2018). The Norwegian Cognitive Impairment After Stroke Study (Nor-COAST): study protocol of a multicentre, prospective cohort study. *BMC Neurol.* 18:193. 10.1186/s12883-018-1198-x 30477436 PMC6260901

[B62] Touroutoglou A. Dickerson B. C. (2019). “Chapter 8-cingulate-centered large-scale networks: normal functions, aging, and neurodegenerative disease,” in *Handbook of Clinical Neurology*, Vol. 166 ed. Vogt B. A. (Amsterdam: Elsevier), 113–127. 10.1016/b978-0-444-64196-0.00008-x 31731908

[B63] van Dalen J. W. van Wanrooij L. L. Moll van Charante E. P. Brayne C. van Gool W. A. Richard E. (2018). Association of apathy with risk of incident dementia: a systematic review and meta-analysis. *JAMA Psychiatry* 75 1012–1021. 10.1001/jamapsychiatry.2018.1877 30027214 PMC6233800

[B64] Vapnik V. (1998). “The support vector method of function estimation,” in *Nonlinear Modeling: Advanced Black-Box Techniques*, eds Suykens J. A. K. Vandewalle J. (Boston, MA: Springer US), 55–85. 10.1007/978-1-4615-5703-6_3

[B65] Wouts L. van Kessel M. Beekman A. T. F. Marijnissen R. M. Oude Voshaar R. C. (2020). Empirical support for the vascular apathy hypothesis: a structured review. *Int. J. Geriatr. Psychiatry* 35 3–11. 10.1002/gps.5217 31617249 PMC6916153

[B66] Wright I. C. McGuire P. K. Poline J. B. Travere J. M. Murray R. M. Frith C. D. (1995). A voxel-based method for the statistical analysis of gray and white matter density applied to schizophrenia. *Neuroimage* 2 244–252. 10.1006/nimg.1995.1032 9343609

[B67] Yushkevich P. A. Piven J. Hazlett H. C. Smith R. G. Ho S. Gee J. C. (2006). User-guided 3D active contour segmentation of anatomical structures: significantly improved efficiency and reliability. *Neuroimage* 31 1116–1128. 10.1016/j.neuroimage.2006.01.015 16545965

[B68] Zi W. Duan D. Zheng J. (2014). Cognitive impairments associated with periventricular white matter hyperintensities are mediated by cortical atrophy. *Acta Neurol. Scand.* 130 178–187. 10.1111/ane.12262 24838230

[B69] Zigmond A. S. Snaith R. P. (1983). The hospital anxiety and depression scale. *Acta Psychiatr. Scand.* 67 361–370. 10.1111/j.1600-0447.1983.tb09716.x 6880820

