# Supplementary material for: Predicting the Emergence of Major Neurocognitive Disorder Within Three Months After a Stroke
Source: Front Aging Neurosci. 2021 Aug 16;13:705889. doi: 10.3389/fnagi.2021.705889 (PMC8418065; doi:10.3389/fnagi.2021.705889)
Supplement: Supplementary file 1 [file Data_Sheet_1.docx]

# Supplementary material

Supplementary table 1: List of medication factor, the condition it is for and the number of patients (out of the final study sample) that have them prescribed at admission to hospital. Second part of table shows the distribution of type of vascular event within the anticoagulant and antiplatelet groups.

Supplementary table 1: List of medication intake factors linked to risk factors for stroke and/or major NCD.

| Medication | Condition | *N* | Type of vascular event | *N* |
| --- | --- | --- | --- | --- |
| *Statins* | High cholesterol | 73 |  |  |
| *Antidiabetic* | Diabetes | 30 |  |  |
| *Antihypertensive* | Hypertension | 112 |  |  |
| *Anticoagulants* | Vascular event | 20 | Cerebrovascular disease | 2 |
|  |  |  | Coronary heart disease | 7 |
| *Antiplatelet* | Vascular event | 82 | Cerebrovascular disease | 36 |
|  |  |  | Coronary heart disease | 39 |

Supplementary table 2: List of MRI sequence parameters across hospitals

| **Hospital** | **Sequence** | **Repetition**  **Time** | **Echo**  **Time** | **Inversion**  **Time** | **Flip**  **Angle** | **Rows** | **Columns** | **FOV** | **Slice**  **Thickness** |
| --- | --- | --- | --- | --- | --- | --- | --- | --- | --- |
| Oslo | T1_BRAVO_iso | 8.16 | 3.18 | 450 | 12 | 256 | 256 | 256x256 | 1 |
|  | CUBE_FLAIR | 8000 | 125.87 | 2092 | 90 | 256 | 256 | 256x256 | 1.2 |
|  | T2-PROPELLER | 6519 | 100.9 | NA | 142 | 512 | 512 | 512x512 | 4 |
|  | SWAN_3D | 37.2 | 23.24 | NA | 10 | 512 | 512 | 512x512 | 2 |
|  | DWI_TETRA | 3500 | 61.2 | NA | 90 | 256 | 256 | 256x256 | 6 |
| St. Olav | T1_MPRAGE_iso | 2300 | 2.01 | 900 | 9 | 256 | 256 | 256x256 | 1 |
|  | 3D_FLAIR | 5000 | 388 | 1800 | 120 | 256 | 256 | 256x256 | 1 |
|  | T2_TSE | 4200 | 81 | NA | 159 | 448 | 448 | 256x256 | 3 |
|  | SWI | 29 | 30 | NA | 15 | 512 | 384 | 512x384 | 2 |
|  | DWI | 6400 | 76 | NA | 90 | 128 | 128 | 128x128 | 4 |
| Haukeland | T1_MPRAGE_iso | 2300 | 2.03 | 900 | 9 | 256 | 256 | 256x256 | 1 |
|  | 3D_FLAIR | 5000 | 386 | 1800 | 120 | 256 | 256 | 256x256 | 1 |
|  | T2 | 4370 | 73 | NA | 150 | 448 | 448 | 448x448 | 3 |
|  | SWI | 29 | 20 | NA | 15 | 512 | 384 | 512x384 | 2 |
|  | DWI | 6400 | 76 | NA | 90 | 130 | 130 | 130x130 | 4 |
| Bærum | T1-3D TFE | 7.47 | 3.44 | NA | 8 | 320 | 320 | 320x320 | 1.1 |
|  | 3D_FLAIR | 4800 | 284.1 | 1660 | 90 | 288 | 288 | 288x288 | 1.14 |
|  | T2 | 7069 | 100 | NA | 90 | 560 | 560 | 560x560 | 4 |
|  | SWI | 51 | 0 | NA | 20 | 672 | 672 | 672x672 | 2 |
|  | DWI | 4773 | 101 | NA | 90 | 224 | 224 | 224x224 | 4 |
| Ålesund | T1_3D MPRAGE (ADNI) | 25 | 4.602 | NA | 30 | 320 | 320 | 320x320 | 1 |
|  | 3D_FLAIR | 4800 | 250.5 | 1660 | 90 | 432 | 432 | 432x432 | 1.32 |
|  | T2 | 5463 | 100 | NA | 90 | 560 | 560 | 560x560 | 5 |
|  | VEN_BOLD | 24.56 | 34.7 | NA | 10 | 512 | 512 | 512x512 | 1 |
|  | DWI | 4623.9 | 108.3 | NA | 90 | 176 | 176 | 176x176 | 4 |

Supplementary table 2: MRI sequences across the five different hospitals. All scanners used T1 3D gradient echo pulse sequences in order to ensure as similar image-quality as possible across hospitals. FOV = Field of view.

Supplementary table 3: Sensitivity measure of model tried by pre-stroke cognitive status

|  | | **Normal cognition + mild NCD + major NCD** | **Normal cognition + mild NCD** | **Normal cognition** |
| --- | --- | --- | --- | --- |
| FACTORS FOR TRAINING | | **Pre-stroke GDS 1-7** | **Pre-stroke GDS 1-3** | **Pre-stroke GDS 1-2** |
|  |  | 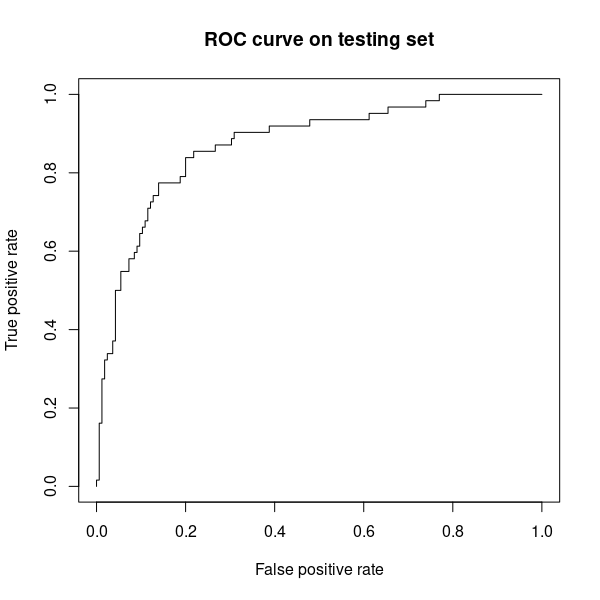  **AUC = .874** | 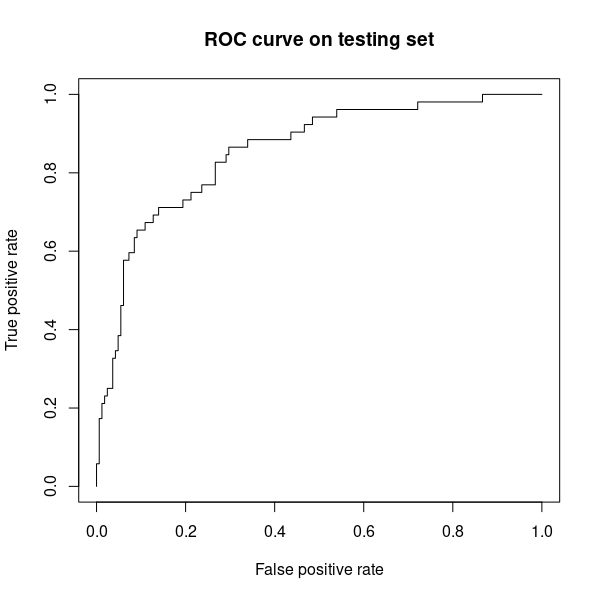  **AUC = .855** | 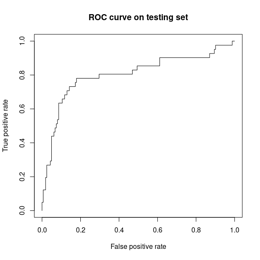  **AUC = .802** |
| *N* |  | **227** | **217** | **203** |
| Age | 1 | Stroke volume | Stroke volume | Stroke volume |
| Gender | 2 | Occipital th. (left) | Occipital th. (left) | Antiplatelets |
| Education (yrs) | 3 | Temporal th. (left) | Antiplatelets | Occipital th. (left) |
| Smoking | 4 | WMH volume | Cingulate th. (right) | Stroke severity^1^ |
| BMI | 5 | Cingulate th. (right) | Temporal th. (left) | Temporal th. (left) |
| Stroke severity ^1^ | 6 | Antiplatelets | Education (years) | Previous infarction |
| Atrial fibrillation | 7 | Education (years) | Stroke severity^1^ | Previous ICH |
| Pre-existing depression | 8 | Stroke severity^1^ | Previous infarction | Education (years) |
| Anticoagulants | 9 | Cingulate th. (left) | Parietal th. (left) | Cingulate th. (right) |
| Statins | 10 | Atrial fibrillation | Previous ICH |  |
| Antidiabetics | 11 | Anticoagulants | Age |  |
| Antihypertensives | 12 | BMI | Frontal th. (left) |  |
| Antiplatelets | 13 | Statins |  |  |
| Comorbidity ^2^ | 14 | Previous ICH |  |  |
| WMH volume | 15 | Frontal th. (left) |  |  |
| Stroke volume | 16 | Gender |  |  |
| Frontal th. (left) | 17 | Frontal th. (right) |  |  |
| Frontal th. (right) | 18 | Comorbidity^2^ |  |  |
| Parietal th. (left) | 19 |  |  |  |
| Parietal th. (right) | 20 |  |  |  |
| Temporal th. (left) | 21 |  |  |  |
| Temporal th. (right) | 22 |  |  |  |
| Occipital th. (left) | 23 |  |  |  |
| Occipital th. (right) | 24 |  |  |  |
| Cingulate th. (left) | 25 |  |  |  |
| Cingulate th. (right) | 26 |  |  |  |
| Previous infarction | 27 |  |  |  |
| Previous ICH | 28 |  |  |  |

Supplementary table 3: Factors listed in descending order according to weight (except in the ‘all factors’-column, which is listed unordered). th. = cortical thickness.^1^ = NIHSS. ^2^ Charlson Comorbidity Index.
